# Immunohistochemical localization of phosphohistidine phosphatase PHPT1 in mouse and human tissues

**DOI:** 10.1080/03009730802642337

**Published:** 2009-04-24

**Authors:** Xiau-Qun Zhang, Ulla Beckman Sundh, Leif Jansson, Örjan Zetterqvist, Pia Ek

**Affiliations:** ^1^Department of Medical Biochemistry and Microbiology, Uppsala UniversityUppsalaSweden; ^2^Toxicology Division, National Food AdministrationUppsalaSweden; ^3^Department of Medical Cellbiology, Uppsala UniversityUppsalaSweden

**Keywords:** Phosphohistidine phosphatase, PHPT1, PHP, phosphohistidine, dephosphorylation, HPR-project

## Abstract

Protein histidine phosphorylation accounts for about 6% of the total protein phosphorylation in eukaryotic cells; still details concerning histidine phosphorylation and dephosphorylation are limited. A mammalian 14-kDa phosphohistidine phosphatase, also denominated PHPT1, was found 6 years ago that provided a new tool in the study of phosphohistidine phosphorylation. The localization of PHPT1 mRNA by Northern blot analysis revealed high expression in heart and skeletal muscle. The main object of the present study was to determine the PHPT1 expression on protein level in mouse tissues in order to get further information on the physiological role of the enzyme. Tissue samples from adult mice and 14.5-day-old mouse embryos were processed for immunostaining using a PHPT1-specific polyclonal antibody. The same antibody was also provided to the Swedish human protein atlas project (HPR) (http://www.proteinatlas.org/index.php). The results from both studies were essentially consistent with the previously reported expression of mRNA of a few human tissues. In addition, several other tissues, including testis, displayed a high protein expression. A salient result of the present investigation was the ubiquitous expression of the PHPT1 protein and its high expression in continuously dividing epithelial cells.

## Introduction

The ability of ATP to donate a phosphate group to amino acid residues in specific proteins under the influence of protein kinases has long been known. The importance of these posttranslational modifications of proteins for regulation of their function has been demonstrated, and phosphorylation is today considered to be one of the most important regulatory mechanism in living organisms. One-third of the proteins found in a typical mammalian cell are phosphorylated at a given time (1). Many human diseases are associated with abnormal phosphorylation of cellular proteins (2). Phosphorylation is regulated by a large number of protein kinases and phosphoprotein phosphatases. The most investigated of these enzymes phosphorylate/dephosphorylate specific serine, threonine, and/or tyrosine residues of a given protein, thereby affecting its function.

Although phosphohistidine represents a significant fraction (6%) of the protein-bound phosphate, eukaryotic histidine phosphorylation has been little studied compared with serine/threonine and tyrosine phosphorylation (3–5). One reason for this is that phosphohistidine is labile under the often very acidic conditions during which protein characterization is performed.

In 1991 an important discovery in histidine phosphorylation was made by the purification of a yeast protein histidine kinase, and since then several reports on histidine protein kinase activity in humans have been reported (5,6). Later a new 14-kDa phosphatase that dephosphorylated phosphohistidine, but neither phosphoserine, -threonine, nor -tyrosine, was purified from porcine liver cytosol and cloned from a human kidney genomic DNA library. It was named phosphohistidine phosphatase (PHPT1), since no phosphoprotein substrates were described at the time (7). This enzyme was independently identified by Klumpp et al. (8) in rabbit liver. The latter group named the enzyme protein histidine phosphatase. The full physiological function of PHPT1 remains to be elucidated, although it has been suggested that the enzyme is involved in the regulation of signalling via G proteins (9) and regulation of ATP-citrate lyase (10). Recently Srivastava et al. (11) described that K^+^ channel KCa3.1 is dephosphorylated by PHPT1, thereby affecting CD4 T cell function that could have an impact on autoimmunity.

PHPT1 has been shown to be rather conserved between species. To increase the knowledge of the role of PHPT1, we have studied, in the present work, the expression of the enzyme on protein level by an immunohistochemical approach. An earlier investigation of a Northern blot of human tissue using a PHPT1 cDNA as probe (7) showed the expression of a 0.6-kb mRNA mainly in the heart and skeletal muscle, whereas the expression in liver was lower. Multiple organs from mouse were included in the present study. Since PHPT1 is homologous to a sex-regulated protein in *Drosophilae*, also testis was included. Organs of mouse embryo from different stages of development were investigated as well. The PHPT1-specific antibodies employed in this study were in parallel used in the Swedish human proteome resource project (HPR project, http://www.proteinatlas.org/index.php). These latter data are also discussed in this paper.

## Materials and methods

### Tissue samples and histological pretreatment

Tissue samples were obtained from adult mice (4 weeks to 8 months old) and E14.5 mouse embryos. The specimens were collected from B6CBAF1 and C57BL/6J mice from Charles River Laboratories (Uppsala, Sweden). All animal experiments had been approved by the Uppsala animal ethics committee. Organs were fixed in 4% paraformaldehyde in 100 mM sodium phosphate/100 mM NaCl, pH 7.5 (PBS). The samples were then embedded in paraffin and sectioned to 6 µm thickness and stored at 4°C. The slides were heated for 30 min at 60°C the same day the immunohistochemistry was performed. Prior to the immunohistochemical treatment the sections were deparaffinized in xylene for 2×5 min and rehydrated in decreasing concentrations of ethanol: 100%, 95%, 70%, for 2×2 min in each concentration, after which the sections were washed twice in PBS for 5 min.

### Antibodies

The primary antibody was raised by Innovagen in rabbit against whole recombinant human PHPT1 that was prepared as described by Ek et al. (7). The rabbit immune serum was affinity-purified against a PHPT1-specific peptide (101–115) bound to CNBr-Sepharose (Innovagen). Peptide 101–115 was chosen since it is essentially identical in several species including mice and humans. Biotinylated goat anti-rabbit immunoglobulin G (IgG), purchased from DAKO, was used as secondary antibody.

### Immunohistochemistry

The slices were proteinase K (Roche)-treated (10 µg/µL in 50 mM Tris/5 mM EDTA, pH 7.5) in order to minimize obstructions that could prevent the binding of the antibody. The tissues were subjected to this treatment for 20 min at room temperature. The proteinase was removed by immersion of the sections in PBS for 2×5 min.

To reduce background staining, additional pretreatment was conducted by immersing the slides in 3% (v/v) H_2_O_2_ in PBS for 5 min at room temperature followed by PBS for 2×2 min.

Thereafter the sections were blocked in a mixture of 3% (w/v) bovine serum albumin (BSA) and 5% (v/v) goat serum in PBS for 30 min at room temperature. The blocking solution was removed by PBS for 2×5 min.

The primary, affinity-purified antibody, with an original concentration of 0.36 mg/L, and diluted in PBS 1/1000 or 1/100, was added, and the slides were incubated at 4°C for 16 h. In absorption control experiments the 1:1000 diluted primary antibody was mixed with 10 µM peptide 101-115 and 3% BSA just before incubation. Preimmune serum was diluted 1/1000 in PBS, when used.

The slides were rapidly warmed at room temperature. After this, the primary antibody was removed with PBS containing 0.1% (v/v) Tween 20 for 5×3 min (shaking). Secondary antibody, diluted 1/1000 in PBS with 3% BSA, was added, and the slides were incubated for 2 h at room temperature. The secondary antibody was then removed with PBS in 0.1% Tween 20 for 5×3 min (shaking).

The slides were incubated at room temperature for 45 min with avidin-biotin-horseradish peroxidase-complex, diluted 1/1000 in PBS. After washing with PBS/0.1% Tween 20 for 5×3 min, the slides were immersed in diaminobenzidine (DAB) (Sigma). The staining process was terminated by removal of DAB, after which the slides were washed in H_2_O or PBS.

The sections were subsequently stained with Mayer's haematoxylin (Bio-Optica) for 2–5 s, rinsed in cold-tap water for 15 min, and dehydrated by immersion in increasing concentrations of ethanol (70%, 95%, 100%) and finally in xylene and mounted with Pertex.

## Results

**Figure 1.**
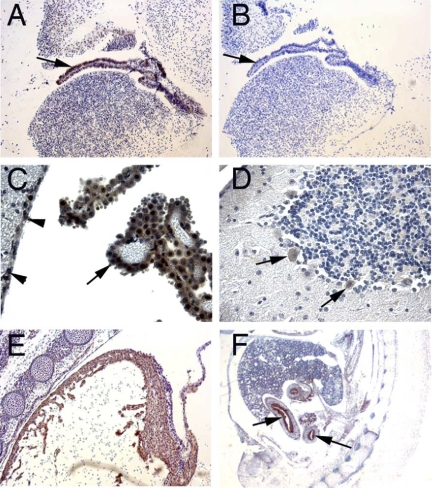
Immunohistochemical staining for PHPT1. A: An E14.5 mouse embryo sagittal section shows PHPT1 signal in the epithelium of the choroid plexus in the fourth ventricle of brain (arrow). B: Adjacent section of A, using preimmune serum as a negative control. C: The same expression pattern of PHPT1 was found in the epithelium layer of the choroid plexus in an adult mouse brain (arrow). Arrowheads point at the ependymal cells in the ventricle. D: Purkinje cell (arrow) in the cerebellum expressing PHPT1. E: PHPT1 expression in the E14.5 embryonic heart muscle. F: PHPT1 expression (arrow) in the epithelium layer of the developing gut of an E14.5 sagittal section. Amplifications were 100× for A, B, and E, 400× for C and D, and 25× for F.

**Figure 2.**
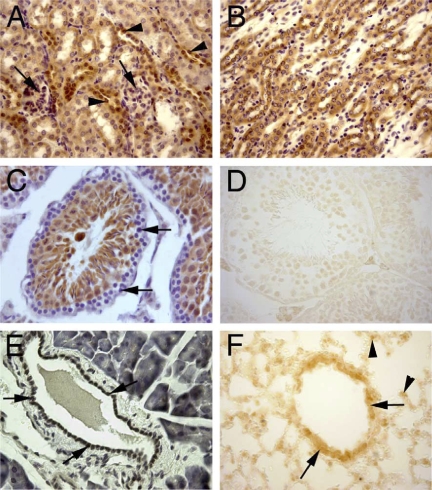
Immunohistochemical staining for PHPT1. A: A section of adult mouse kidney shows PHPT1 expression in the distal convoluted tubules (arrowhead) but not in the glomeruli (arrow) and a weak expression in the proximal convoluted tubule. B: PHPT1 is expressed in the Henle's loops of adult kidney. C and D: Sections of seminiferous tubule of adult mouse testis. Arrows in C point to the spermatogonium of seminiferous tubule. D: An absorption test shows that PHPT1 signals were abolished in the mouse testis. E: PHPT1 is expressed in the epithelium of interlobular duct of pancreas (arrow). F: PHPT1 is expressed in the epithelium of bronchiole (arrow). Arrowheads point at macrophages in alveoli. Amplifications were 400× for A–F.

In this work, the protein expression of phosphohistidine phosphatase, PHPT1, was investigated by immunohistochemistry, using an affinity-purified polyclonal PHPT1 antibody on sections of paraffin-embedded mouse tissues. Samples were taken from both E14.5 embryos and adult mice of the C57BL/6 J or B6CBAF1 strains. Concomitantly, the antibodies used in the present study were included in the HPR project, where expression of proteins in normal and cancerous human tissues and cell lines is determined. Some of the mouse results are illustrated in Figures 1 and 2, and the human results can be found at http://www.proteinatlas.org/index.php. The mouse tissue sections were examined, and the staining intensity was ranked from (−), for no staining, to (+++) for a strong signal. In the case of mouse tissues, the results were confirmed in at least two additional series using tissues from different mice. The PHPT1 protein showed a tissue-specific expression pattern in mouse and human tissues, and the results are summarized in Table I.

**Table I.** Protein expression of PHPT1 in mouse and human tissues.

Name of tissues	Mouse E14.5	Mouse adult	Human adult
Brain			
Choroid plexus epithelium	+++	+++	ND
Ependymal cells of ventricles	ND	+++	ND
Cells in the hippocampus	ND	++	ND
Cerebral cortex			
Glia cells	ND	−	−
Neuronal cells	ND	−/+	+
Cerebellum			
Cells in granular layer	ND	−/+	−
Cells in molecular layer	ND	−	−
Purkinje cells	ND	+/++	+
Intestine and stomach			
Surface epithelium	+++	+	++
Smooth muscle	++	++	ND
Liver			
Bile duct epithelium	ND	++	−/+
Hepatocytes	++	+	++
Kupffer cells	−	−	ND
Pancreas			
Interlobular duct epithelium	++	++	+
Islet cells	ND	+	+
Acinar cells	−	+	++
Spleen			
Cells in red pulp	ND	−/+	+
Cells in white pulp	ND	+/ −	+
Lung			
Alveolar epithelium	−	−	−
Alveolar macrophages	ND	++	++
Epithelium of bronchioles and bronchi	+	++	+
Testis			
Seminiferous tubules			
Spermatogonia	ND	−	++
Spermatocytes	ND	++	++
Spermatids	ND	++	+
Interstitial cells	ND	−	++
Epididymis			
Ductal epithelium	ND	++	ND
Kidney			
Cells in glomeruli	ND	−	−
Cells in tubules			
Proximal tubules	ND	−/+	++
Distal tubules	ND	++	+
Henle's loops	ND	++	ND
Collecting tubules	ND	−/+	ND
Adrenal glands			
Cortical cells	ND	+/++ ^a^	++
Medullar cells	ND	−/+	ND
Bone and cartilage	+	ND	ND
Epidermal cells	+	ND	+++
Muscle			
Heart muscle	++	+/++	++
Skeletal muscle	++	+/++	++
Smooth muscle of vessels	ND	+	+

^a^Positive cells are found within the zona glomerulosa. ND = not determined; − = no signal; + = weak signal; ++ = moderate signal; +++ = strong signal.

### Specificity of the PHPT1 antibody

To evaluate the specificity of the PHPT1 antibody, a peptide absorption test was performed in which PHPT1 peptide 101-115 was added to the PHPT1 antibody before incubation with the sections. The absorption test dramatically reduced the immunoreactive signals. An example is seen in Figure 2D, when compared with Figure 2C. Preimmune sera were, in several experiments, used in parallel, and no staining was obtained. An example is seen in Figure 1B. These results confirmed that the binding of the antibody to the PHPT1 peptide was specific.

### PHPT1 expression in mouse embryo

In the mouse E14.5 sections, strong PHPT1 immunostaining was seen in the epithelium of choroid plexus of brain ventricles and at the surface epithelium of the gastrointestinal tract (Figure 1 and Table I). The embryonic heart muscle (Figure 1E) and skeletal muscle also expressed PHPT1, while the control sections incubated with preimmune serum did not demonstrate any staining (Figure 1B).

### PHPT1 expression in adult tissues

*Brain*. In the adult mouse brain, PHPT1 was specifically expressed in the epithelium layer of the choroid plexus, while the mesenchymal tissue of choroid plexus did not exhibit any expression (Figure 1C). In cerebellum, the Purkinje cells clearly showed PHPT1 signals also in the nuclei (Figure 1D). There were also small groups of cells scattered in the mid-brain and hippocampus regions that were stained for PHPT1 (data not shown). The identity of these latter cell types is not yet clear. In the cerebral cortex of adult mouse, no cells that demonstrated clear PHPT1 expression were seen.

*Gastrointestinal tract including liver*. Weak to moderate level of PHPT1 expression was seen in the gastrointestinal surface epithelium of the digestive tract, both in adult mouse and in human (Table I). PHPT1 was clearly expressed in the epithelium of mouse ducts of internal organs. A moderate PHPT1 expression was seen in the bile duct epithelium of the adult mouse liver, whereas a weak signal was seen in the hepatocytes (data not shown). The Kupffer cells and endothelial cells of liver did not stain for PHPT1. In adult mouse pancreas, the epithelium of interlobular ducts specifically and strongly expressed PHPT1, while the mouse acinar cells showed no or only a weak signal (Figure 2E). The human acinar cells, on the other hand, displayed a signal at a moderate level. The islet endocrine cells weakly expressed PHPT1 in the adult mouse and moderately so in the human tissue (Table I).

*Lung*. In the adult mouse lung, the capillary endothelial cells of alveolar septa stained negatively for PHPT1. Alveolar macrophages in the alveoli expressed PHPT1 with a weak to moderate signal. The epithelium of bronchioles and bronchi also expressed PHPT1 (Figure 2F).

*Testis*. No staining was observed in the spermatogonia located on the basement membrane surrounding the seminiferous tubules, whereas the labelling of spermatocytes and spermatids (that is, later stages of spermatogenesis) was moderate (Table I, Figure 2C). Interestingly, in human testis, spermatogonia moderately expressed PHPT1 with a somewhat lower expression in the spermatocytes and a weak expression in spermatids. Further, human interstitial cells of Leydig moderately expressed PHPT1, which was in contrast to those in adult mouse testis (see interstitial cells in Table I). In mouse, the epithelium of the duct of the epididymis showed moderate PHPT1 expression (Table I).

*Kidney*. PHPT1 expression in adult mouse kidney was mainly restricted to the cortex. PHPT1 was not expressed in any parts of the glomeruli (Figure 2A). It was very weakly expressed in the epithelium of the proximal tubuli, but was clearly and strongly expressed in the epithelium of Henle's loop and distal tubule (Figure 2A and B). However, the strong expression of PHPT1 was dramatically decreased, or even lacking, in the collecting tubuli in the medulla. No or weak expression of PHPT1 was seen in the renal papilla and calyx.

*Muscle*. PHPT1 was also expressed in striated muscle of adult mouse, including both skeletal and heart muscle. The smooth muscle of the gastrointestinal tract and blood vessels also expressed PHPT1 (Table I).

### Overview and intraspecies comparison

A comparison between the PHPT1 immunohistochemical staining results in mouse and human tissues revealed a similar expression pattern in the majority of tissues, e.g. strong expression in the epithelium and a moderate expression level in heart and sceletal muscle cells and some glandular cells.

The PHPT1 expression in different epithelial tissues was strong both in adult and embryonic mouse. PHPT1 was detected in the simple columnar epithelial tissue that covers the digestive tubules (such as intestine and stomach), the pseudostratified columnar ciliated epithelium of the lung bronchia, and the simple cuboidal epithelium of kidney tubules (Figure 2A and B and Table I). PHPT1 was also clearly detected in both stratified and non-stratified squamous epithelium in e.g. adult human skin and oesophagus. PHPT1 was not detectable in most simple, squamous epithelial cells, such as endothelium and alveolar epithelium, which were all negative. Developing muscle also expressed PHPT1, such as the heart muscle (Figure 1E) and gut smooth muscle at embryonal stage E14.5, but a relatively lower expression level was found in the corresponding adult tissues.

However, differences in the expression level between mouse and human were also found. Thus in the bile duct epithelium PHPT1 was more strongly expressed in mouse than in human tissue, while the opposite was the case in pancreatic acinar cells (Table I). In the adrenal gland PHPT1 was only found in cells of the zona glomerulosa in mouse but was homogenously expressed throughout the cortex in human tissue. Different expression patterns between the species were also seen for spermatogonia of testis and the epithelium of proximal convoluted tubules of kidney, as outlined above (Table I).

## Discussion

In two independent studies (7,8) a 14-kDa phosphohistidine phosphatase (PHPT1) was isolated and characterized. Later studies on the enzyme have suggested that its substrates include a subunit of heterotrimeric G proteins (Gβ) (9) and ATP-citrate lyase (10). However, as the turn-over rate for these substrates seems to be much lower than for the phosphohistidine peptide, used by Ek et al. (7), the identification of true physiological substrates may still remain. During the preparation of the present manuscript, Srivastava et al. (11) published the finding that the calcium-activated K^+^ channel KCa3.1, phosphorylated on a histidine residue by nucleoside diphosphate kinase beta (NDPK-B), was dephosphorylated by PHPT1 which caused a decrease in the K^+^ channel activity in CD4 T cells. However, whether KCa3.1 is a main physiological substrate of PHPT1, or only one of several physiological substrates, remains to be established.

In the present work, we selected an immunohistochemical approach in an attempt to further characterize the role of PHPT1. A screening of a large number of tissues for the localization of the enzyme protein was considered to give independent hints as to the function of the enzyme. Such a study also made possible the comparison of the mRNA expression (7) and the protein expression of PHPT1.

PHPT1 protein was expressed in all organs tested, even though the extent varied. In order to eliminate possible mistakes in the interpretation of results, the specificity of the primary antibody was carefully tested using both preimmune serum and depletion assays as controls. This confirmed that the antibodies were specific, since no staining for PHPT1 was seen after the antibody had been saturated with the PHPT1 peptide 101–115 prior to being added to the tissue (Figure 2D) or when preimmune serum was used (Figure 1B).

There was a fairly good correlation between protein expression and the expression level of mRNA. Similar results are found in the PubMed UniGene ESTProfile database. It must be emphasized, however, that the comparison was made between organ-specific cell expression of PHPT1 protein and whole-organ expression of PHPT1 mRNA.

Protein kinases and phosphatases are often involved in cell proliferation. It is therefore noteworthy that PHPT1 expression was strong in varying types of rapidly dividing epithelial tissues but not detectable in most of the endothelium, a normally quiescent epithelium, in both species tested and that all human cancers of ectodermal origin showed moderate PHPT1 expression (HPR homepage). Another example of PHPT1 being involved in a proliferation context is a recurrent 9q34 duplication in paediatric T cell acute lymphoblastic leukaemia that was found in 33% of the cases with increased mRNA expression of the PHPT1 gene (12). In the HPR database, the relative PHPT1 protein expression level was moderate, 8%, in the only human leukaemia cell line analysed, but the presence or absence of duplication in this particular cell line is not known. Further, the expression of PHPT1 is increased upon *ex vivo* radiation exposure of blood cells, as are CDKN1A, FDXR, SESN1, and BBC3, known possible regulators in cell proliferation or apoptosis (13). Additionally, in the PubMed UniGene ESTProfile database, glioma cells have high expression of PHPT1 mRNA, which correlates to the HPR finding for malignant glioma and glioblastoma cell lines, where the immunostaining was moderate to strong. Normal glia cells in both adult mouse and human did not express PHPT1. In view of these findings we suggest that PHPT1 might have a role during cell proliferation and differentiation.

The results from the immunohistochemical staining of PHPT1 in human tissues were essentially similar to mouse PHPT1 expression patterns. The strongest signal was found in the squamous epithelial cells of skin, oesophagus, vagina, and cervix. However, some tissues showed expression level differences between the two species or even contradictory results. The reasons for these differences are not clear. They may be due to true species differences or due to difference in tissue preparation or other methodological differences between the laboratories.

A partial homology to the sex-regulated protein Jan A in *Drosophilae* prompted us to investigate the expression of PHPT1 in the reproductive organs. The protein had a moderate level of expression in both human and mouse testis and in the human uterus epithelium (not determined in mouse). In the PubMed UniGene ESTProfile database, mRNA expression is high also in the mammary gland, but in this database there are also organs that have even higher levels of mRNA expression, e.g. heart, pituitary gland, and parathyroid glands. A specific role for the enzyme in sex regulation is therefore not obvious.

The staining profile of ATP-citrate lyase, reported to be a substrate of PHPT1 (10), did not overlap that of PHPT1 in human normal tissue, cancer cells, and cell lines, as can be seen on the HPR home page. The beta subunit of G protein, reported to be colocalized with and dephosphorylated by PHPT1 (9), is not represented in the HPR database and can therefore not be compared with the staining profile of PHPT1. This is also the case for the calcium-activated K^+^ channel KCa3.1 Since this channel and PHPT1 could be coimmunoprecipitated in the CD4 T cells (11), the localization of at least a part of the PHPT1 protein, as studied in the present work, may coincide with that of KCa3.1. However, the resolution of the immunohistochemical method used does not allow for an unequivocal discrimination between a PHPT1 signal from the plasma membrane and from other subcellular structures except for a clear expression in nuclei found for some hepatocytes (data not given) and for all Purkinje cells (Figure 1D). Furthermore, if KCa3.1 only represents one of several substrates of PHPT1 the signal from the channel-bound enzyme may be weak compared to all other signals.

In conclusion, in the present immunochemical study the expression of PHPT1 has been investigated in mouse embryo and adult mouse organs. At the same time, in the HPR project, the PHPT1 expression in human tissues, cancers, and cell lines has been investigated. The expression in various tissues was essentially similar in the two species. Of particular interest was the finding that tissues where the epithelial cells have a short half-life were strongly stained, which suggests that PHPT1 is involved in cell proliferation.
